# Safety, Tolerability, Pharmacokinetics, and Pharmacodynamics of Cholic Acid (MT921) after a Subcutaneous Injection in the Submental Area to Humans

**DOI:** 10.3390/ph14080830

**Published:** 2021-08-23

**Authors:** Hyewon Chung, Jin-Woo Park, Dai-Hyun Kim, Soo-Hong Seo, Kyoung-Ah Kim, Woo-Shun Lee, Ji-Young Park

**Affiliations:** 1 Department of Clinical Pharmacology and Toxicology, Korea University Guro Hospital, Seoul 08308, Korea; hyewonchung@korea.ac.kr; 2 Department of Clinical Pharmacology and Toxicology, Korea University College of Medicine, Korea University Anam Hospital, Seoul 02841, Korea; parkzinu@korea.ac.kr (J.-W.P.); kakim920@kumc.or.kr (K.-A.K.); 3 Department of Neurology, Anam Hospital, Korea University Medical Center, Seoul 02841, Korea; 4 Department of Dermatology, College of Medicine, Korea University, Seoul 02841, Korea; mightycell@naver.com (D.-H.K.); drsshong@korea.ac.kr (S.-H.S.); 5 Medytox Inc., Seoul 06175, Korea; drlee@medy-tox.co.kr

**Keywords:** cholic acid, pharmacokinetics, pharmacodynamics, clinical trial

## Abstract

This study aimed to explore pharmacokinetics, pharmacodynamics, and safety/tolerability of MT921, an injectable cholic acid, after a single subcutaneous administration to healthy volunteers. A randomized, double-blinded, placebo-controlled, single dose-ascending phase 1 study enrolled 24 subjects who were assigned to three groups (60 mg, 120 mg, and 150 mg) of MT921. Blood samples were obtained for a 24-h period before and after injecting MT921 to the submental fat area. Plasma concentrations of cholic acid and deoxycholic acid were determined for pharmacokinetic analysis. Levels of free fatty acid, triglyceride, and total cholesterol were measured for pharmacodynamic analysis. Safety and tolerability were assessed until 21 days post-dose. While systemic exposure to cholic acid tended to increase as the MT921 dose increased, pharmacokinetic profiles of deoxycholic acid were similar among dose groups without showing significant changes. Pharmacodynamic profiles were comparable when measured at baseline and post-dose. The most frequent adverse events were injection site pain and edema. All adverse drug reactions resolved without treatment. MT921 appeared to be well-tolerated after an injection to the submental area at a dose up to 150 mg. Systemic exposure to cholic acid increased as the dose increased. Blood lipid profiles and deoxycholic acid levels were not affected by MT921 treatment.

## 1. Introduction

The submental line is one of the criteria that determines a youthful neck [1]. Due to the aging process, obesity, and genetic factors, undesirable neck contour by submental fat can appear [2]. From an aesthetic point of view, it can lead to negative feelings and behaviors such as wearing clothing to conceal the neck or avoiding video chats [3]. A survey on cosmetic dermatologic procedures collected from 3645 consumers has reported that 73% of them are bothered by excess fat under the chin and neck [4]. In the United States, 63,993 procedures of injection lipolysis were conducted in 2018 with a total expenditure of more than USD 67 million, showing a rising trend [5].

Traditionally, surgical options such as liposuction have been used for submental fat reduction. However, minimally invasive treatments are desired because surgical options need a substantial period for recovery with possible complications after surgery [6]. Deoxycholic acid was approved in 2015 as a pharmacological agent in the United States of America for improving the appearance of moderate to severe convexity, or fullness, associated with submental fat in adults [7]. Clinical trials have reported that it can significantly reduce submental fat measured by clinician-reported and patient-reported rating scales as well as magnetic resonance imaging [8,9].

Cholic acid is a primary bile acid with a molecular weight of 408.6. Together with chenodeoxycholic acid in humans, they are synthesized from cholesterol in hepatocytes. These exist largely as glycine and taurine conjugates stored in the gall bladder. After conjugated cholic acid is secreted into the intestine, it is deconjugated and converted to deoxycholic acid. Most bile acids are reabsorbed at the terminal ileum. Deoxycholic acid is reabsorbed in the colon and recycled to undergo enterohepatic circulation [10].

Recently, oral dosages of cholic acid were approved for use in the treatment of bile acid synthesis disorders due to single enzyme defects or for adjunctive treatment of peroxisomal disorders [11]. To date, injectable cholic acid has not been approved for medical use. One study has shown that deoxycholic acid removes more protein, membrane enzymes and phospholipids than cholic acid [12]. Thus, provided that cholic acid injection brings adequate clinical efficacy, it might be a drug of choice for reducing submental fat with less cytotoxicity than deoxycholic acid.

MT921 is a pharmaceutical product that is under development. MT921 has been developed as a liquid injection with a pH of 7.4 containing a buffer and salts with 1.5% cholic acid as the main ingredient. Cholic acid has both hydrophilic and hydrophobic properties by adding a hydrophilic group to cholesterol, which enables cholic acid to act as a surfactant. Because all biological cell membranes have the same lipid bilayer structure, injecting MT921 is expected to exhibit its pharmacological activity by cell lysis.

The aim of this study was to explore pharmacokinetics (PK), pharmacodynamics (PD), and safety/tolerability of MT921 in healthy adults after a single administration to submental fat.

## 2. Results

### 2.1. Subjects

A total of 25 subjects were enrolled. One subject withdrew his consent before the investigational product administration. Thus, 24 subjects were injected with MT921 or the placebo and completed the study. All subjects were males. Their age, height, weight, and body mass index were not significantly different among dose groups (Table 1).

### 2.2. Pharmacokinetics of Cholic Acid and Deoxycholic Acid

**Figure 1:**
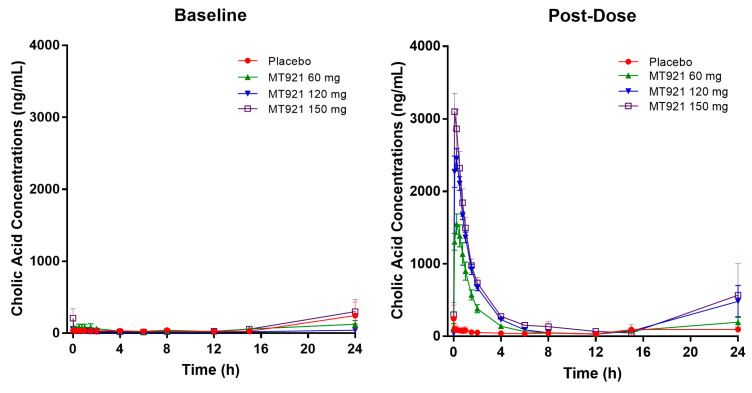
Mean plasma concentration-time profile of cholic acid before and after administration of MT921.

**Figure 2:**
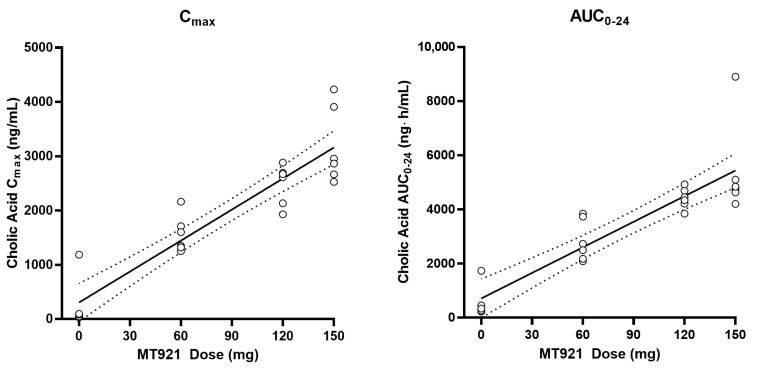
Pharmacokinetic dose-proportionality assessment of cholic acid after administration of MT921.

**Figure 3:**
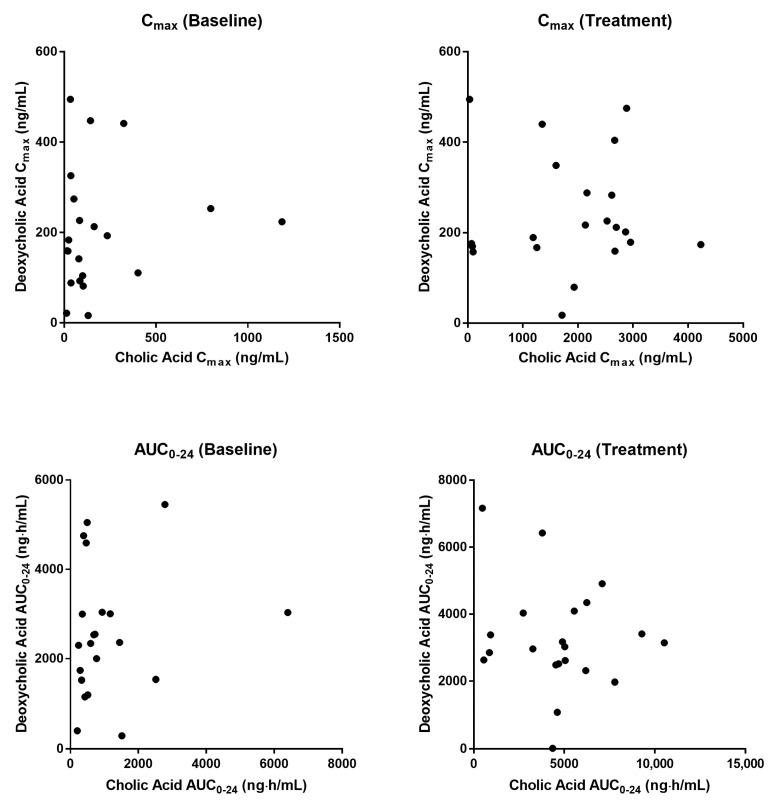
Relationship of pharmacokinetic parameters between cholic acid and deoxycholic acid.

Baseline pharmacokinetic profiles were similar among dose groups, although basal cholic acid exhibited high inter-individual variability, with a range of 36-fold in C_max_ and 9-fold in AUC. When MT921 was administered to the submental area subcutaneously, systemic cholic acid level reached T_max_ before 0.5 h post-dose and returned to baseline at approximately 6 to 8 h post-dose (Figure 1, Table 2). Systemic exposure to cholic acid increased as the dose of MT921 increased, while that to deoxycholic acid was similar without showing significant differences among dose groups (Supplementary Figure S1, Table 2). The power model for baseline adjusted cholic acid revealed less than proportional characteristics for C_max_, while the dose-proportionality of AUC_0-24_ could not be concluded. R_dnm_ (90% CI) for C_max_ and AUC_0-24_ were 0.7773 (0.6082–0.9935) and 0.9755 (0.5031–1.8912), respectively (Figure 2). There was no relationship between PK parameters of cholic acid and deoxycholic acid at baseline or after MT921 injection (Figure 3).

### 2.3. Pharmacodynamics

**Figure 4:**
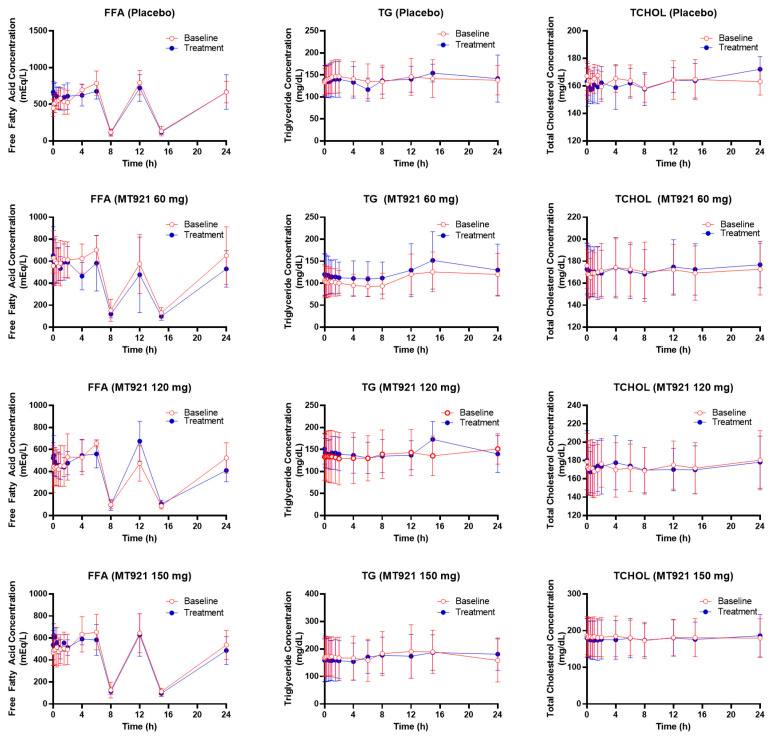
Mean plasma concentration-time profile of free fatty acid (FFA), triglyceride (TG), and total cholesterol (TCHOL) before and after administration of MT921.

Mean concentration–time profiles of free fatty acid, triglyceride, and total cholesterol were comparable when measured at baseline and post-dose regardless of the dose group (Figure 4). There were no statistically significant differences in PD parameters among dose groups either (Table 3).

### 2.4. Safety and Tolerability

Among the 24 subjects who were administered with either the placebo or MT921, 19 subjects reported 55 adverse events. There were no serious or severe adverse events. Except for one case of ligament sprain, which was considered to be unrelated to the investigational product; all other adverse events were local irritability occurring at the administration site and were resolved without treatment. The most frequent adverse events were injection site pain and edema. All subjects who were administered MT921 reported at least one adverse event (Table 4). There was no clinically meaningful finding on physical examination, vital signs, or clinical laboratory tests.

## 3. Discussion

To our knowledge, this is the first report on a clinical study of MT921 composed of cholic acid to provide the results such as safety, tolerability and pharmacokinetic profiles along with baseline-adjustments as well as information from the metabolic product, deoxycholic acid, in humans. It has been revealed that the pharmacokinetics of MT921 might not be influenced by co-administered drugs such as amlodipine, simvastatin, and pioglitazone using in vitro and in silico experiments [13].

In this study, the PK of MT921 developed for reducing submental fat was characterized. Cholic acid is an endogenous compound derived from cholesterol [14]. Thus, basal cholic acid levels before MT921 treatment were also assessed. Systemic exposure of cholic acid was substantially elevated after MT921 dosing. Its increments were dose linear. C_max_ and AUC values of cholic acid for the group receiving MT921 150 mg were 3193 ng/mL and 8344 ng∙h/mL, respectively, higher than those of the placebo group (319 ng/mL and 1618 ng∙h/mL, respectively). This suggested that even though MT921 was injected locally to the submental area, cholic acid from MT921 was absorbed into the blood stream. Nonetheless, considering the higher variability of basal cholic acid levels, its systemic effect is expected to be minor. We did not find any systemic adverse event related to the treatment in this study.

The basal AUC values of cholic acid ranged 200–6938 ng∙h/mL in this study’s groups. This highly variable inter-individual baseline may have led to a comparable baseline adjusted AUC_0-24_ between MT921 120 mg and 150 mg dose groups although their C_max_ values were dose-dependent.

We found that plasma cholic acid level increased at 24 h which was around 8 a.m. regardless of the treatment. Bile acid homeostasis is known to be regulated by the circadian rhythm in humans [15]. Although the mechanism is unclear, our finding is consistent with a previous publication which showed high basal unconjugated bile acid levels at late night to early morning in healthy subjects [16]. Increased pools of cholic acid reaching enterohepatic circulation after the injection of MT921 may have led to dose-dependent elevation of cholic acid at 24 h.

Deoxycholic acid is a metabolite formed from cholic acid [17]. Thus, its levels in plasma were also assessed in this study. Baseline profiles of deoxycholic acid showed high variability, similarly to cholic acid. Its levels in the blood increased after taking a meal, similarly to the previous data [18,19]. Blood levels of deoxycholic acid after MT921 injection did not differ from those obtained from basal assessment, indicating no metabolic conversion of deoxycholic acid from the subcutaneously injected cholic acid. Because deoxycholic acid is usually formed from cholic acid by microbiota in the colon [17], the formation of deoxycholic acid after MT921 subcutaneous injection might not have occurred.

This study was a phase 1 study which mainly aims to explore safety, tolerability, and the pharmacokinetic profile of the study drug in healthy participants. As the study design was not intended to observe a clinical effect on reducing submental fat, we additionally explored pharmacodynamics of MT921 as systemic changes in lipid profiles. Cholic acid is one of bile acids synthesized from cholesterol by 7-α-hydroxylase whose transcription is suppressed by primary bile acids but increased by bile acid binding resin cholestid or cholesterol [20]. It has been reported that blood cholesterol level decreased by 15% to 20% after intake of deoxycholic acid in healthy volunteers and patients with gallstones [21,22]. Although systemic exposure to cholic acid was observed after MT921 injection to the submental area, lipid profiles after MT921 treatment were comparable to those obtained from baseline measurement without showing any statistically significant difference, suggesting that local injection of MT921 might play a minor role in the change of lipid profiles.

All treatment-related adverse events were localized at the injection site. They were mild or moderate in severity. They did not require any medical treatment. In addition, no clinically meaningful change in laboratory findings or vital signs from baseline values was observed. All participants treated with MT921 reported injection site edema and pain. These were predictable considering that cholic acid has detergent characteristics that can cause inflammation [12]. The most frequent adverse events observed in this study were edema, local pain, bruising, and numbness. The adverse events were consistent with those after injection of deoxycholic acid which can be used to reduce submental fat clinically [23,24]. Taken together, MT921 was well tolerated by subjects. It exhibited a good safety profile in this study without causing any serious adverse events.

## 4. Materials and Methods

### 4.1. Subjects

Written informed consents were obtained from all subjects prior to this study. Healthy subjects aged 20 to 65 years with enough submental fat to inject the investigational product were enrolled. Subjects who had clinically significant disease, allergy, or medical history were excluded. Subjects who had abnormality or surgical history for the administration site and subjects whose triglyceride or total cholesterol levels exceeded their normal limits were also excluded.

### 4.2. Study Design

This was a randomized, double-blinded, placebo-controlled, single center, single dose-ascending phase 1 study (Clinicaltrials.gov identifier: NCT03905291). It was performed in compliance with the declaration of Helsinki, good clinical practice, and local regulation. It was approved by the Institutional Review Board (IRB) of Korea University Anam Hospital, Seoul, Korea. (Approval number: 2018AN0119; Approval date: 11 May 2018).

Eight subjects were enrolled for each dose group. Of them, six were randomized to receive MT921 and two were randomized to receive the placebo. A total of three doses (60 mg, 120 mg, and 150 mg) of MT921 were used in this study. Dose escalation was based on the safety profile until the 6th day of administration of the prior dose group.

Eligible subjects were admitted to Korea University Anam Hospital 2 days before administration of the investigational product. On the 2nd day of admission, blood samples were drawn to obtain baseline PK/PD characteristics. On the morning of the 3rd day after admission, MT921 at 60 mg, 120 mg, or 150 mg, or normal saline was administered to each subject’s submental fat area at fasting state. Each subcutaneous injection containing 0.2 mL of MT921 (3 mg), or normal saline was separated from the adjacent injection with a distance of 1 cm. Blood sampling for PK/PD analysis was performed at pre-dose and at 5, 15, 30, 45 min, and at 1, 1.5, 2, 4, 6, 8, 12, 15, and 24 h post-dose. Lunch and dinner were provided at 6 and 12 h post-dose, respectively. Time points for blood sampling and meals remained same for the baseline assessment.

Subjects were discharged on the day after the injection. Safety and tolerability were assessed until 21 days post-dose based on reported adverse events, administration site local tolerability, physical exam, vital signs, clinical laboratory tests, and an electrocardiogram.

### 4.3. Determination of Plasma Concentrations of Cholic Acid and Deoxycholic Acid

After blood samples were centrifuged at 3000 rpm for 15 min at 4 °C, plasma samples were obtained and kept frozen below −60 °C until use. Finally, plasma samples were placed on dry ice and sent to Biocore Co., Ltd. (Seoul, Korea) for analysis. Blood levels of cholic acid and deoxycholic acid were determined using a high-performance liquid chromatography (Shimadzu UFLC, Shimadzu, Japan) coupled with a tandem mass spectrometry (QTRAP 5500, AB Sciex, Framingham, MA, USA). Plasma samples of 100 μL were mixed with 400 μL acetonitrile and 10 μL of internal standard working solution containing 1000 ng/mL cholic acid-d5 and 1000 ng/mL deoxycholic acid-d5. After centrifuging at 13,000 rpm for 5 min, the organic layer was evaporated for 15 min at 45 °C and then reconstituted using 200 μL of 50% acetonitrile. After the solution was centrifuged at 13,000 rpm for 5 min, 2 μL of supernatant was injected to a C18 column (2 × 75 mm, particle size 3 μL) at 40 °C. The mobile phase consisted of 10 mM ammonium acetate and acetonitrile at 70:30 (*v*/*v*) with a flow rate of 0.3 mL/min. The mass spectrometer was operated in negative ion mode using multiple reaction monitoring. The mass transition ion pairs were selected as *m*/*z* 407.3 → 407.3, *m*/*z* 412.3 → 412.3, *m*/*z* 391.3 → 391.3, and *m*/*z* 396.3 → 396.3 for cholic acid, cholic acid-d5, deoxycholic acid, and deoxycholic acid-d5, respectively.

The calibration curve was linear for 5–2000 ng/mL of cholic acid and 10–4000 ng/mL of deoxycholic acid. Intra study accuracy was 95.5–104.3% and 93.2–102.7% and precision was 1.0–6.5% and 0.8–3.7% for cholic acid and deoxycholic acid, respectively.

### 4.4. Pharmacokinetic/Pharmacodynamic Analysis

The non-compartmental method implemented in Phoenix WinNonlin^®^ version 7.0 (Pharsight, CA, USA) was used for PK/PD analysis. For PK analysis, maximum concentration (C_max_) and time to reach C_max_ (T_max_) were directly determined from individual time-concentration profiles. The area under the concentration-time curve from dosing to 24 h post-dose (AUC_0-24_) was then calculated. Baseline-adjusted PK parameters were derived by subtracting parameters measured at baseline from parameters measured post-dose. For PD analysis, levels of free fatty acid, triglyceride, and total cholesterol were measured. Their maximum effects (E_max_) were directly determined from the individual profiles and areas under the effect-time curve (AUEC) were then calculated using the linear trapezoidal method.

### 4.5. Statistical Analysis

All statistical analyses were carried out using SAS^®^ 9.4 (SAS Institute Inc., Cary, NC, USA). Descriptive statistics were used to summarize demographics, PK/PD parameters, and adverse events. Demographic data and PD parameters were compared between dose groups using analysis of variance (ANOVA) and a *p*-value less than 0.05 was considered to be statistically significant. Power model was used to assess PK dose-proportionality among dose groups. Dose-proportionality was concluded when the 90% confidence interval (CI) for the ratio of dose-normalized model-predicted geometric mean values for high and low doses (R_dnm_) was within the range of 0.8–1.25 [25].

## 5. Conclusions

In conclusion, MT921 appeared to be safe and well tolerated after a local injection into the submental area at dosages up to 150 mg. The PK of cholic acid was well characterized. Its systemic exposure increased dose-linearly. However, blood lipid profiles and deoxycholic acid levels were not affected by MT921 treatment.

## Figures and Tables

**Table 1:** Demographic summary of subjects.

	Placebo (*N* = 6)	60 mg (*N* = 6)	120 mg (*N* = 6)	150 mg (*N* = 6)	Total (*N* = 24)
Age (years)	26.7 ± 3.7	34.7 ± 12.0	32.7 ± 11.4	27.8 ± 4.4	30.5 ± 8.9
Height (cm)	172.5 ± 5.3	172.8 ± 5.7	175.7 ± 5.5	172.0 ± 4.0	173.3 ± 5.0
Weight (kg)	83.57 ± 6.65	81.30 ± 10.38	81.92 ± 8.14	83.23 ± 5.88	82.50 ± 7.47
BMI (kg/m^2^)	28.05 ± 1.10	27.11 ± 2.11	26.48 ± 1.18	28.12 ± 1.39	27.44 ± 1.56

Data are presented as mean ± standard deviation.

**Table 2:** Pharmacokinetic parameters of cholic acid and deoxycholic acid by dose group.

Analyte	Parameter	Placebo (*N* = 6)	60 mg (*N* = 6)	120 mg (*N* = 6)	150 mg (*N* = 6)
Cholic Acid	T_max_ (h)	0.04 (0–15)	0.25 (0.08–0.5)	0.25 (0.08–0.25)	0.08 (0.08–0.25)
	C_max_ (ng/mL)	319 ± 451	1568 ± 342	2488 ± 370	3193 ± 703
	Baseline adjusted C_max_ (ng/mL)	70 ± 156	1413 ± 291	2400 ± 342	2769 ± 663
	AUC_0-24_ (ng∙h/mL)	1618 ± 1501	4262 ± 1185	7071 ± 2505	8344 ± 6851
	Baseline adjusted AUC_0-24_ (ng∙h/mL)	−67 ± 2141	2887 ± 1108	6582 ± 2344	6319 ± 4498
Deoxycholic Acid	T_max_ (h)	8 (0–12)	24 (0.25–24)	24 (24–24)	24 (0.08–24)
	C_max_ (ng/mL)	237 ± 144	252 ± 164	237 ± 135	237 ± 96
	Baseline adjusted C_max_ (ng/mL)	−35 ± 46	57 ± 147	64 ± 205	62 ± 53
	AUC_0-24_ (ng∙h/mL)	4009 ± 1844	3383 ± 2329	2701 ± 1077	3065 ± 1062
	Baseline adjusted AUC_0-24_ (ng∙h/mL)	869 ± 922	586 ± 1220	424 ± 917	970 ± 798

Data are presented as mean ± standard deviation, except for T_max_ which is presented as median (min–max).

**Table 3:** Pharmacodynamic parameters by dose group.

Analyte	Parameter	Placebo (*N* = 6)	MT921 60 mg (*N* = 6)	MT921 120 mg (*N* = 6)	MT921 150 mg (*N* = 6)
Free Fatty Acid	E_max_ (mEq/L)	844.50 ± 126.98	832.00 ± 287.24	738.67 ± 158.42	732.17 ± 132.42
	AUEC (mEq∙h/L)	11,010.61 ± 2183.47	8846.40 ± 2277.39	8739.81 ± 1093.95	9255.14 ± 1385.66
	Baseline adjusted E_max_ (mEq/L)	2.00 ± 132.58	31.17 ± 217.58	41.67 ± 161.63	−36.50 ± 197.70
	Baseline adjusted AUEC (mEq∙h/L)	−487.36 ± 1314.18	−1799.47 ± 2301.01	106.26 ± 1158.29	−532.02 ± 2078.38
Triglyceride	E_max_ (mg/dL)	167.67 ± 39.50	153.83 ± 65.63	178.00 ± 40.8	200.50 ± 67.64
	AUEC (mg∙h/dL)	3381.84 ± 704.86	3069.19 ± 1217.15	3508.22 ± 857.22	4201.19 ± 1576.94
	Baseline adjusted E_max_ (mg/dL)	10.17 ± 25.79	22.67 ± 27.62	15.17 ± 17.37	−11.17 ± 30.11
	Baseline adjusted AUEC (mg∙h/dL)	10.51 ± 271.19	390.99 ± 469.07	181.05 ± 430.11	−28.40 ± 402.08
Total Cholesterol	E_max_ (mg/dL)	174.50 ± 10.69	181.50 ± 24.80	184.00 ± 29.71	190.33 ± 57.99
	AUEC (mg∙h/dL)	3929.37 ± 249.66	4146.27 ± 548.96	4144.37 ± 629.21	4279.70 ± 1219.62
	Baseline adjusted E_max_ (mg/dL)	1.50 ± 10.56	1.67 ± 7.89	−1.50 ± 4.85	−0.33 ± 10.25
	Baseline adjusted AUEC (mg∙h/dL)	10.07 ± 94.89	36.32 ± 241.13	−18.95 ± 90.91	−42.69 ± 156.79

Data are presented as mean ± standard deviation.

**Table 4:** Number of subjects and duration of treatment-emergent adverse events.

Adverse Event	Placebo	MT921 60 mg	MT921 120 mg	MT921 150 mg
Injection Site Bruising	-	[2] 5.5 (5–6)	[5] 18 (6–20)	[2] 14 (8–20)
Injection Site Erythema	-	[1] 1 (1–1)	[1] 2 (2–2)	[3] 2 (2–2)
Injection Site Hypoesthesia	-	[1] 9 (9–9)	[1] 62 (62–62)	-
Injection Site Nodule	-	[1] 17 (17–17)	-	-
Injection Site Edema	[1] 3 (3–3)	[6] 5 (4–5)	[6] 16.5 (6–36)	[6] 23 (8–27)
Injection Site Pain	-	[6] 1.5 (1–4)	[6] 11 (1–36)	[6] 20 (8–23)

Data are presented as [Number of subjects] and median (min–max) of duration as days.

## Data Availability

The data presented in this study are available on request from the corresponding author, upon reasonable request. The data are not publicly available due to the privacy issue.

## Supplementary Materials

The following are available online at https://www.mdpi.com/article/10.3390/ph14080830/s1, Supplementary Figure S1. Mean plasma concentration-time profile of deoxycholic acid before and after administration of MT921.
