# Supplementary material for: Safety, Tolerability, Pharmacokinetics, and Pharmacodynamics of Cholic Acid (MT921) after a Subcutaneous Injection in the Submental Area to Humans
Source: Pharmaceuticals (Basel). 2021 Aug 23;14(8):830. doi: 10.3390/ph14080830 (PMC8400465; doi:10.3390/ph14080830)
Supplement: Supplementary file 1 [file pharmaceuticals-14-00830-s001.zip › pharmaceuticals-1297845-supplementary.pdf]

pharmaceuticals

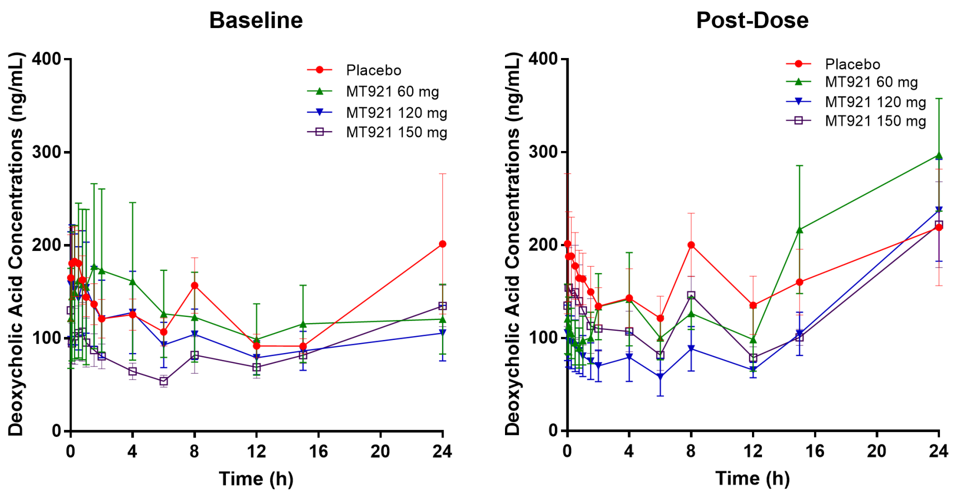

Supplementary Figure S1. Mean plasma concentration-time profile of deoxycholic acid before and after administration of MT921.
